# Immunization Associated with Erectile Dysfunction Based on Cross-Sectional and Genetic Analyses

**DOI:** 10.1371/journal.pone.0111269

**Published:** 2014-10-24

**Authors:** Yang Chen, Xianxiang Xin, Haiying Zhang, Jianfeng Xu, Yong Gao, Aihua Tan, Xiaobo Yang, Xue Qin, Yanling Hu, Zengnan Mo

**Affiliations:** 1 Center for Genomic and Personalized Medicine, Guangxi Medical University, Nanning, Guangxi Zhuang Autonomous Region, China Zhuang Autonomous Region, China; 2 Institute of Urology and Nephrology, First Affiliated Hospital of Guangxi Medical University, Nanning, Guangxi Zhuang Autonomous Region, China; 3 Department of Occupational Health and Environmental Health, School of Public Health of Guangxi Medical University, Nanning, Guangxi Zhuang Autonomous Region, China; 4 Department of Clinical Laboratory, First Affiliated Hospital of Guangxi Medical University, Nanning, Guangxi Zhuang Autonomous Region, China; 5 Medical Scientific Research Centre, Guangxi Medical University, Nanning, Guangxi Zhuang Autonomous Region, China; 6 Fudan Institute of Urology, Huashan Hospital, Fudan University, Shanghai, China; 7 Fudan Center for Genetic Epidemiology, School of Life Sciences, Fudan University, Shanghai, China; 8 State Key Laboratory of Genetic Engineering, School of Life Sciences, Fudan University, Shanghai, China; 9 Center for Cancer Genomics, Wake Forest University School of Medicine, Winston-Salem, North Carolina, United States of America; Midwestern University, United States of America

## Abstract

Erectile dysfunction (ED) is a global disease affecting a large number of people. Some studies have found a relationship between low-grade inflammation and ED. We hypothesized that the immune system might play a key role in the outcome of ED. Five immune agents (C3, C4, IgA, IgM, and IgG) were collected based on the Fangchenggang Area Male Health and Examination Survey (FAMHES), using methods of a traditional cross-sectional analysis. Our results repeated the significant association between ED and metabolic syndrome, obesity, and so forth. However, there seemed to be no positive relation between the tested indexes and ED risk in the baseline analysis (C3: *P* = 0.737; C4: *P* = 0.274; IgA: *P* = 0.943; IgG: *P* = 0.069; IgM: *P* = 0.985). Then, after adjusting for age and multivariate covariates, a potentially significant association between ED and IgG was discovered (*P* = 0.025 and *P* = 0.034, respectively). Meanwhile, in order to describe the development of ED on a gene level, SNP–set kernel-machine association test (SKAT) was applied with the known humoral immune genes involved. The outcomes suggested that PTAFR (binary *P* value: 0.0096; continuous *P* value: 0.00869), IL27 (0.0029; 0.1954), CD37 (0.0248; 0.5196), CD40 (0.7146; 0.0413), IL7R (0.1223; 0.0222), PSMB9 (0.1237; 0.0212), and CXCR3 (0.0849; 0.0478) might be key genes in ED, especially IL27, when we restricted the family-wise error rate (FWER) to 0.5. Our study shows that IgG and seven genes (PTAFR, CD37, CD40, IL7R, PSMB9, CXCR3, and especially IL27) might be key factors in the pathogenesis of ED, which could pave the way for future gene and immune therapies.

## Introduction

Erectile dysfunction (ED) is also known as inadequate penile erection and mainly affects men older than 40; it is defined as the inability to acquire and maintain satisfying sexual intercourse with a sufficient erection [1], [2]. It is said that the clinical disorder was described in ancient Egypt more than 5000 years ago [3]. ED constitutes a substantial burden on public health and has high prevalence. Among men younger than 40, the prevalence of ED is 1–10%. In men aged above 40, the morbidity is considerably increased (2–9% for the age of 40–49 years old, and 20–40% for 60–69; 50–100% for those older than 70) [4]–[8]. A general practitioner is estimated to treat 1–2 new cases of ED per month [2]. It has been predicted that the number of cases of ED worldwide might reach 322 million by the year 2025 [9], [10]. Therefore, the discovery and management of the factors influencing the occurrence of ED are important.

According to recent studies, ED is potentially associated with many diseases. In 2013, Weinberg et al. [11] investigated the association between the severity of diabetes, metabolic syndrome, and ED risk using a cross-sectional analysis, which indicated significant associations among them. Among male patients with type 1 or 2 diabetes mellitus, 35–75% had ED, whereas, in the population without diabetes, 5–15% had ED [12], [13]. In addition, epidemiological studies found that ED might be one of the markers of cardiovascular disease (CAD). Kumar et al. [14] and Nehra et al. [15] showed that patients with CAD frequently had ED and that the symptoms of ED always preceded those of CAD. Therefore, treatment of ED should be considered secondary to decreasing cardiovascular risk. Although the relationships between ED risk and diabetes mellitus, hypertension, hyperlipidemia, metabolic syndrome, depression, and lower urinary tract symptoms have been investigated, the role of the immune system in ED has been neglected.

In 2006, Blans et al. [16] studied the associations of ED in patients with diabetes, infections, and inflammation, and found that elevated high-sensitivity C-reactive protein and infections might play a role in ED among patients with diabetes. Furthermore, Araña Rosaínz et al. [17] found a potential correlation between ED and low-grade inflammation. With the association between inflammation, infection, and ED risk, we hypothesized that the development and progression of ED might be associated with the immune system to a significant extent. In order to identify the factors in ED risk, this cross-sectional study was conducted on humoral immunity function indexes based on FAMHES [18]. In addition, further gene-based analysis was conducted by collecting the genes for humoral immunity in order to present comprehensive associations between immune cytokines and ED risk. This study sheds new light on the functions of immune factors in ED among males.

## Methods and Materials

### Study population

All samples in this study were collected from the Fangchenggang Area Male Health and Examination Survey (FAMHES), which was mainly focused on environmental and genetic factors, as well as their interrelations. As a population-based study conducted among non-institutionalized Chinese men aged from 17 to 88 years old in Guangxi, FAMHES investigated the development of age-related chronic disease. However, after completing the study, we removed juvenile subjects and ensured that only data from adult participants (age ≥18 years) were included in this analysis. In the comprehensive demographic and health survey, 4303 men participated in routine physical examination at the Medical Centre in Fangchenggang First People’s Hospital from September to December 2009. Then, data were collected from 3593 of the participants in the form of interviews. The response rate was 83.5% [18]. There were no distinct differences between the men who participated in the interviews and those who did not. Written informed consent was obtained for all participants. The study was approved by the medical ethics committee of Guangxi Medical University.

The analysis included five immune parameters [complement 3 (C3), complement 4 (C4), immunoglobulin A (IgA), immunoglobulin G (IgG) and immunoglobulin M (IgM)], each of which was analyzed as an independent factor in order to explore the association between them and ED risk. When screening the data, we applied the following exclusion criteria: (1) low-value immune parameters (IgA, IgG, IgM, C3, and C4) or refusing to contribute blood serum; (2) incomplete information on the individual; (3) did not complete the ED questionnaire; (4) currently suffering from myocardial infarction, congestive heart failure, stroke, hyperthyroidism, rheumatoid arthritis, acquired immune deficiency syndrome, or any kind of cancer, or having a history of pelvic or urinary tract trauma/surgery/inflammation or chronic hepatitis; (5) immune parameters beyond the normal threshold values (acute infection or immunocompromised), according to our local laboratory standards (normal ranges IgA 0.7–3.5 g/L, IgM 0.5–2.6 g/L, IgG 7.0–16.6 g/L, C3 0.8–1.5 g/L, and C4 0.2–0.6 g/L); (6) at present, taking medicines that could influence ED and immunity such as psychotropic drugs, non-steroidal anti-inflammatory drugs, antibiotics, spironolactone, cimetidine, glucocorticoids or other steroidal drugs, and so on.

Finally, 1205 participants were included in the analysis for complement C3 (577 ED patients and 628 non-ED patients). For complement C4, 1288 participants were included (611 for ED and 677 for non-ED). There were 1188 eligible participants for the analysis of IgA (572 for ED and 616 for non-ED). In addition, 1243 participants (590 ED and 653 non-ED) were studied for IgG. Lastly, for IgM, 1233 participants were included (586 for ED and 647 for non-ED).

### Phenotypes and covariates

The known evaluation system of the International Index of Erectile Function (IIEF-5) was applied to define ED in the study [19]. Five items were assessed (erection confidence, erection firmness, maintenance ability, maintenance frequency, and satisfaction). The IIEF-5 scores range from 5 to 25. For each item, five questions are asked, each with a score of 5 points. Decreasing scores indicate poorer sexual function and increased severity of ED. According to the criteria, the scores were divided into five categories to define ED status: none (IIEF-5 score 22–25); mild (17–21); moderate (12–16); and severe (5–11). In addition, a cut-off point of an IIEF-5 score of 22 was used for the dichotomous variable (ED and non-ED) [20], [21].

A comprehensive questionnaire was applied in the survey, which was completed during a face-to-face interview. Collection of essential information (age, sex, smoking, drinking, and so on) and complete physical examinations (height, weight, waistline, hipline, etc.) were conducted by trained personnel using a standardized protocol. Smoking status was defined as never smoked, former smoker (cessation of smoking), and current smoker [22]. Alcohol consumption was defined as consumption of alcohol including beer, wine, and hard liquor in the lifetime of the participant. Meanwhile, the participants were divided into three groups, according to the number of years of education (0–6, 7–9, and ≥10 years).

For the physical examination, body weight with thin clothing and height without shoes were measured. The body mass index (BMI) was categorized into three groups (normal weight <24.0 kg/m^2^, overweight 24.0–27.9 kg/m^2^, obese 28.0 kg/m^2^). The waist circumference was measured at the midpoint between the inferior costal margin and the superior iliac crest on the mid-axillary line. The hipline was defined as the maximum circumference over the buttocks. The waist-hip ratio (WHR) was categorized as normal weight (WHR ≤0.9) and obese (WHR>0.9) [23]. Additionally, based on the metabolic syndrome, the following indexes were also defined: hypertriglyceridemia, triglycerides ≥1.7 mmol/L; low high-density lipoprotein cholesterol (HDL-C), HDL-C ≤1.03 mmol/L; elevated blood pressure (BP), BP≥130/85 mmHg; hyperglycemia, fasting glucose ≥5.6 mmol/L [24], [25].

### Serum measurements

Blood samples were collected from participants, who had been fasting for at least 8 h (overnight), between 8∶00 to 11∶00 in the morning. The samples were transported within 2–3 h to the testing center of the Department of Clinical Laboratory at the First Affiliated Hospital of Guangxi Medical University in Nanning, and were centrifuged within 15–25 min and stored at −80°C. In the Department of Clinical Laboratory at Fangchenggang First People’s Hospital, serum triglycerides, HDL-C, and glucose were measured. The C3, C4, IgA, IgM, and IgG levels were detected with an electrochemiluminescence immunoassay on the COBAS 6000 system E601 (Elecsys module) immunoassay analyzer (Roche Diagnostics, GmbH, Mannheim, Germany) with the same batch of reagents. All details about the laboratory tests can be found in our previous publications [18], [25].

### Single nucleotide polymorphism (SNP) genotyping

All of the genotype data used in our analysis were from the samples of our previous stage 1 genome-wide association analysis (GWAS) using the Illumina Omni 1 platform [26]. The polymerase chain reaction and extension primers were designed by Mass MRRAY Assay Design 3.1 software (Sequenom, Inc.). We performed all genotyping reactions in 384-well plates. Each plate included a duplicate for three or four subjects, selected at random, as well as six-to-nine negative controls, in which water was used instead of DNA. The average concordance rate was 99.8%.

### Statistical analysis

All continuous variables were tested with the Shapiro-Wilks test. Considering the markedly skewed distribution, the immune items (C4, IgA, IgM, and IgG) were logarithmically transformed in the subsequent analysis. According to the IIEF-5, a score of 22 was the cut-off for ED and non-ED, the values of which were presented as two compared groups. The Mann-Whitney *U* test and *X^2^* test were applied where appropriate. Then, the binary logistic and linear regression models were applied in order to assess the association between ED risk and the immune items after adjusting for the covariates. Three groups (unadjusted, age-adjusted, and multivariate-adjusted models) were applied in both the linear regression and binary logistic regression. The covariates in the multivariate-adjusted models were as follows: age; smoking status; alcohol consumption; BMI; and WHR. All analyses were performed with SPSS version 16.0 software (SPSS Inc., Chicago, IL, USA). The statistical tests were two-tailed.

After the cross-sectional analysis, a potential association was found for IgG and ED risk. In order to preliminarily discover the relationship between immune risk and ED risk, further analysis was conducted, attempting to discover the significant immune-related genes, especially for humoral immunity, associated with ED risk, which will give us new insights into the therapy for ED and further potential research avenues. In this analysis, data on 106 genes associated with humoral immunity were collected from the database for genes and proteins of the human immune system [27]. Then, representative tag SNPs were selected among the various loci in these genes with SNPinfo (http://manticore.niehs.nih.gov/snpinfo/snptag.htm) [28], in which tag SNPs were defined as the common SNPs (minor allele frequency [MAF] ≥0.05; *r* ^2^≥0.8) among Asians (CHD+CHB+JPT). Finally, a total of 1266 tag SNPs were involved. All genotypes of the tag SNPs were collected with the Plink [29]. When conducting the analysis, we applied the SNP-set kernel-machine association test (SKAT) [30]. In 2010, along with the development of GWAS, many disease-related genetic factors had been discovered. However, standard analysis of this approach suffered from limited reproducibility and difficulties in detecting multi-SNP and epistatic effects. On the basis of this condition, the team of Lin et al. proposed the method of SKAT analysis, which was mainly based on a logistic kernel-machine model. With that the joint effect of the SNPs in a given SNP set was under consideration, this method provided a total *P*-value for the set of variants tested by aggregating individual score test statistics of the SNPs. Every gene containing a series of tag SNPs was treated as an SNP set. After adjusting for age, association analysis was conducted for ED risk and for the genes. In addition, multiple tests were used on the basis of the null hypothesis of no association with a set of SNPs or a SNP, which provided the empirical distribution of statistical tests. Then, a family-wise error rate (FWER) was evaluated using 1000 permutations with three different cut-offs (0.05, 0.5, and 1). All *P*-values were two-sided and calculated with R v3.0.1.

## Results

As a prevalent disease, ED affects a large population of males around the world. Recently, several studies have reported that many factors, such as diabetes mellitus, metabolic syndrome, depression, and so on, are significantly associated with ED risk [11], [14]. Meanwhile, some studies have suggested that low-grade inflammation might play a key role in the pathogenesis of ED [16], [17]. Therefore, we hypothesized that there might be key genes and immune substances influencing the risk of ED. Based on Fangchenggang Area Male Health and Examination Survey (FAMHES), which was mainly focused on population health, a cross-sectional analysis was conducted on five immune-related agents [complement 3 (C3), complement 4 (C4), immunoglobulin A (IgA), immunoglobulin G (IgG) and immunoglobulin M (IgM)]. Baseline characteristics of the 1243 samples in the analysis are shown in Table 1. The results show that IgG could potentially be associated with ED risk, after adjusting for age. Meanwhile, in this analysis, a significant association between ED and metabolic syndrome, obesity, and so on was again identified [31]. In addition, seven genes [platelet-activating factor receptor (PTAFR), interleukin 27 (IL27), CD37 molecule (CD37), CD40 molecule (CD40), interleukin 7 receptor (IL7R), proteasome subunit, beta type, 9 (PSMB9), and chemokine (C-X-C motif) receptor 3 (CXCR3)] related to humoral immunity, which might be vital in inducing ED, were analyzed with SKAT. This study might shed new light on the influence of the immune system and immune genes on ED risk.

**Table 1:** Characteristics of the eligible samples in this analysis for IgG and erectile dysfunction.

	Total	ED	NO-ED	P value
		≤21	>21	
Number	1243	590	653	
Age	35.34±9.37	37.35±10.56	33.53±7.71	**<0.001**
lnIgG (mg/L)	2.54±0.16	2.55±0.16	2.53±0.17	0.069
BMI (kg/m^2^)	23.44±3.43	23.59±3.29	23.30±3.55	0.126
<24		328	400	
<28, ≥24		210	187	
≥28		52	66	**0.031**
WHR (%)				
≤0.9		355	426	
>0.9		235	227	0.065
Smoking (%)				
Never		255	274	
Former		22	17	
Current		313	362	0.429
Drinking (%)				
No		85	64	
Yes		505	589	**0.013**
Education (%)				
0–6		11	2	
7–9		128	84	
≥10		451	567	**<0.001**
Hypertriglyceridemia		161	199	0.216
Elevated BP		66	77	0.738
Low HDL-C		43	60	0.225
Hyperglycemia		156	130	**0.006**

*IgG was logarithmically transformed in the following analysis.

*IgG = Immunoglobulin G, ED = Erectile dysfunction, IIEF = International Index of Erectile Function, BMI = Body Mass Index, WHR = Waist Hip Rate.

*Student's t test and Analysis of Variance analysis were applied for (ANOVA) quantitative traits; Chi square test (x2) was for the qualitative traits.

### Cross-sectional analysis of association

In this study, five items (C3, C4, IgA, IgG, and IgM) were included in the analysis of ED risk. Significant differences were found in the groups, according to age (C3: *P*<0.001; C4: *P*<0.001; IgA: *P*<0.001; IgG: *P*<0.001; IgM: *P*<0.001), BMI (C3: *P* = 0.023; IgG: *P* = 0.031), WHR (C3: *P* = 0.015), alcohol consumption (C3: *P* = 0.004; C4: *P* = 0.007; IgA: *P* = 0.012; IgG: *P* = 0.013; IgM: *P* = 0.006), education status (C3: *P*<0.001; C4: *P*<0.001; IgA: *P*<0.001; IgG: *P*<0.001; IgM: *P*<0.001), and hyperglycemia (C3: *P* = 0.001; C4: *P* = 0.002; IgA: *P* = 0.002; IgG: *P* = 0.006; IgM: *P* = 0.003) (results not shown). However, there seems to be no positive association between the tested indexes and ED in the baseline analysis (C3: *P* = 0.737; C4: *P* = 0.274; IgA: *P* = 0.943; IgG: *P* = 0.069; IgM: *P* = 0.985). Considering the crucial influence of age and other factors, we conducted both linear regression and binary logistic regression, with the following covariates: age; smoking status; alcohol consumption; BMI; and WHR. In the linear regression, there was no obvious positive association presented when the IIEF-5 score of ED was treated as a dependent variable. However, to our surprise, a significant relationship between ED (IIEF-5≤21) and IgG was discovered after adjusting for age (OR = 0.446, 95%CI: 0.221–0.902, *P* = 0.025) in the subsequent binary logistic regression analysis. Meanwhile, when we conducted multivariate adjustments, the same positive relationship was found (OR = 0.459, 95%CI: 0.223–0.944, *P* = 0.034). In addition, there seemed to be no correlation between the other items (C3, C4, IgA, and IgM) and ED risk. All results are shown in Tables 2 and 3.

**Table 2:** Association between immune substances and IIEF-5 score of ED in the multivariate regression analysis.

IIEF-5 scores															
		IgA			IgG			IgM			C3			C4	
	Beta	95%CI	P	Beta	95%CI	P	Beta	95%CI	P	Beta	95%CI	P	Beta	95%CI	P
Unadjusted	0.015	−0.594, 1.008	0.612	−0.031	−2.246, 0.625	0.268	0.012	−0.477, 0.742	0.670	0.027	−0.744, 2.084	0.353	0.000	−1.006, 0.993	0.990
Age-adjusted	0.027	−0.416, 1.171	0.351	−0.040	−2.448, 0.392	0.156	0.000	−0.613, 0.592	0.973	0.041	−0.370, 2.425	0.150	0.017	−0.683, 1.287	0.548
Multivariate adjusted	0.034	−0.331, 1.276	0.249	−0.034	−2.328, 0.580	0.239	0.003	−0.573, 0.641	0.913	0.018	−1.084, 1.992	0.562	−0.002	−1.061, 0.981	0.939

* Multivariate adjusted for age, smoking status, alcoholic drinking, BMI, WHR.

* As a continuous variable, IIEF-5 score was treated as dependent variable analyzed by linear regression.

* IIEF-5 = 5-item International Index of Erectile Function; BMI = Body Mass Index; WHR = waist hip rate.

**Table 3:** Association between immune substances and ED in the multivariate logistic regression analysis.

Erectile dysfunction (ED)															
		IgA			IgG			IgM			C3			C4	
	OR	95%CI	P	OR	95%CI	P	OR	95%CI	P	OR	95%CI	P	OR	95%CI	P
Unadjusted	0.986	0.678, 1.435	0.943	0.529	0.267, 1.050	0.069	0.997	0.748, 1.330	0.985	1.122	0.573, 2.198	0.736	0.768	0.479, 1.232	0.274
Age-adjusted	1.103	0.751, 1.619	0.619	**0.446**	**0.221, 0.902**	**0.025**	0.908	0.675, 1.221	0.523	1.422	0.711, 2.846	0.320	0.916	0.563, 1.492	0.725
Multivariate adjusted	1.099	0.744, 1.624	0.634	**0.459**	**0.223, 0.944**	**0.034**	0.930	0.689, 1.254	0.633	1.590	0.739, 3.420	0.236	0.860	0.518, 1.427	0.559

* Multivariate adjusted for age, smoking status, alcoholic drinking, BMI, WHR.

* As a dichotomous variable, ED was treated as dependent variable with binary logistic regression applied.

* BMI = Body Mass Index; WHR = waist hip rate.

**Figure 1:**
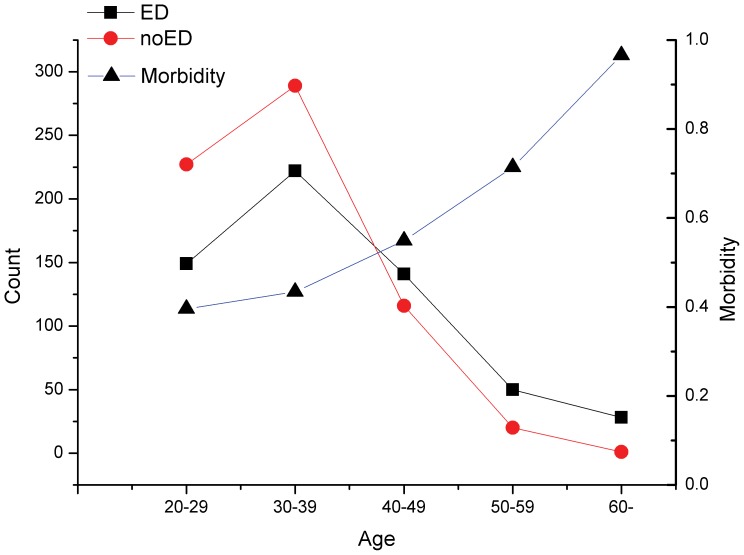
The tendency of the morbidity of erectile dysfunction (ED) along with age growth. * X-axis stands for ages with 10 years range difference. * There are two Y-axes. On the left, the axis stands for the counts of ED or no-ED patients. As for the right one, it represents the morbidity of ED in different age brackets.

Recently, some studies have presented that the morbidity of ED was increasing with increasing age [4]–[8]. This time, in order to test this hypothesis, we tried to describe the tendency of morbidity as a function of age on the basis of data for IgG analysis. In this analysis, age was divided into five groups (18–29, 30–39, 40–49, 50–59 and 60+). The number of patients and morbidity of ED are presented in Figure 1. The results confirmed the hypothesis that the morbidity of ED was related to age. Meanwhile, the increasing rate was enhanced when the age exceeded approximately 40.

### Immune-related genes are associated with ED

According to the IIEF-5 score and the cut-off (i.e. a score of 22), the phenotypes were divided into continuous and binary variants. On the basis of the available database, 106 genes related to humoral immunity were analyzed. Every set of tag SNPs for genes were entered into further SKAT analysis. The results showed that PTAFR (binary IIEF-5: *P* = 0.0096; continuous IIEF-5: *P* = 0.00869), IL27 (binary IIEF-5: *P* = 0.0029; continuous IIEF-5: *P* = 0.1954), CD37 (binary IIEF-5: *P* = 0.0248; continuous IIEF-5: *P* = 0.5196), CD40 (binary IIEF-5: *P* = 0.7146; continuous IIEF-5: *P* = 0.0413), IL7R (binary IIEF-5: *P* = 0.1223; continuous IIEF-5: *P* = 0.0222), PSMB9 (binary IIEF-5: *P* = 0.1237; continuous IIEF-5: *P* = 0.0212), and CXCR3 (binary IIEF-5: *P* = 0.0849; continuous IIEF-5: *P* = 0.0478) could be key genes in influencing ED (Table 4). When calculating the FWER with 1000 permutations, we divided them by three different cut-off points (0.05, 0.5, and 1). There were no positive genes when the FWER was restricted to 0.05. When FWER was restricted to 0.5, IL27 was shown to be associated significantly with ED (binary IIEF-5: *P* = 0.0022; Table 5).

**Table 4:** The significant association for the genes of humoral immunity and ED.

Gene	SNP. Test	Binary	Continuous
PTAFR	2	**0.0096**	0.0869
IL27	1	**0.0029**	0.1954
CD37	1	**0.0248**	0.5196
CD40	3	0.7146	**0.0413**
IL7R	8	0.1223	**0.0222**
PSMB9	5	0.1237	**0.0212**
CXCR3	1	0.0849	**0.0478**

* Phenotypes were divided into two groups: Binary group (ED & no-ED) and Continuous group (defined by the IIEF).

* The SNP. Test was the number of SNPs analyzed in the associated test.

**Table 5:** By comparing the P-value of each test to the distribution of the minimum P-values obtaining from 1000 permuted data sets Family wise error rate (FWER) was applied with the SKAT in binary and continuous group.

	FWER = 0.05	FWER = 0.5	FWER = 1
Binary			
IL27	–	0.0022	0.0022
PTAFR	–	–	0.0113
CD37	–	–	0.0403
AIRE	–	–	0.0516
DEFA6	–	–	0.0396
Continuous			
RFXANK	–	–	0.0653
CD40	–	–	0.0425
IL7R	–	–	0.0136
PSMB9	–	–	0.0566
CXCR3	–	–	0.0577

* The FWER was defined as three cut off (0.05, 0.5, 1).

## Discussion

ED affects a large number of people worldwide. However, not many researches have investigated the relationship between the immune system and ED. This study was conducted to explore the association between immune substances, key genes, and risk of suffering from ED. The results suggest that IgG and seven genes (PTAFR, IL27, CD37, CD40, IL7R, PSMB9, and CXCR3) could be key factors in the pathogenesis of ED, which are potential targets for immune and gene therapy and, therefore, the subject of future studies.

### Immune substances associated with ED

This cross-sectional analysis of five immune elements (C3, C4, IgA, IgG, and IgM) and ED risk was conducted based on a population health survey. In the essential analysis, we found that age, obesity, drinking, educational status, and hyperglycemia could be associated significantly with ED risk, as shown in recent studies [31]–[33]. In order to discover the tendency of morbidity with age, we presented the relationship by intuitively drawing a curve chart. The results confirmed the significant association between ED morbidity and age increase. On the other hand, it also identified that the morbidity increases obviously when the age is above 40 years old, which had been confirmed previously in other studies [4]–[8]. In addition, our results suggest that the level of blood glucose might also be positively associated with ED risk. This conclusion was also verified by related research, after comprehensive reading recent studies. In 2014, Shi et al. [34] suggested diabetes mellitus markers of ED, providing an opportunity for early intervention in aboriginal adult males. At the same time, in one cross-sectional study among Turkish patients, Cander et al. [35] confirmed a significant association between ED and diabetes mellitus. For educational status, we also identified a distinct difference between the three groups with differing numbers of years in education (0–6, 7–9, and ≥10 years). In 2014, Ettala et al. [36] studied the relationships between the risk factors and ED, which identified that a higher education level might decrease the risk of ED. So, according to our baseline analysis, we proposed that age, educational status, body weight, alcohol consumption, and level of blood glucose might be the risk factors influencing ED development.

However, the five immune agents that we investigated showed no positive relationships with ED risk. Among the agents, IgG may be a potential factor for ED risk, as the *P-*value was close to being significant (*P* = 0.069). In order to examine the real relevance of IgG in ED, we adjusted for other influencing factors. A significant association emerged between IgG and ED after adjusting for age (*P* = 0.025) and multivariate covariates (*P* = 0.034). This result not only indicates a relationship between IgG and ED risk, but it also highlights the importance of age, with the *P*-value increasing after multivariate adjustment. In 2006, Blans et al. [16] investigated the relationships between infection, inflammation, and ED. The results indicated that inflammation and related agents played a crucial role in ED risk. When an infection induced an immune response, the concentrations of circulating inflammatory markers would increase, which, in turn, resulted in diffuse changes in the vessel wall by influencing the endothelial function [37], [38]. In addition, Yin et al. [39] provided a new therapy of ED by injecting nerve-injury-induced protein 1 antibody (Ninj1-Ab) in mice, which showed promising efficacy. Therefore, we suggest that immune therapy could be a promising method of treating ED.

### Genes associated with ED

From the available database, data on genes related to humoral immunity were collected. After analysis by SKAT, *P*-values of the genes were used to estimate the association between the genes and ED risk. Seven genes (PTAFR, IL27, CD37, CD40, IL7R, PSMB9, and CXCR3) seemed to show a positive association with ED. The genes are known to be associated significantly with the immune system and inflammatory reaction. PTAFR encodes a seven-transmembrane G-protein-coupled receptor for platelet-activating factor (PAF), which plays a role in oncogenic transformation, tumor growth, angiogenesis, metastasis, and pro-inflammatory processes [40]–[42]. In 2013, Predescu et al. [43] confirmed the effects of PAF in activating endothelial nitric oxide synthase (NOS), which plays a key role in the human corpus cavernosum smooth muscle cell relaxation process [44]. Additionally, the protein encoded by the IL27 gene is one of the subunits of a heterodimeric cytokine complex that plays a key role in B-cell differentiation and functions [45]. As a cytokine, IL27 may also be important in inflammation [46], [47]. However, a relationship between IL27 and the pathogenesis of ED has not been reported. Another gene, CD37, encodes the leukocyte antigen CD37, which mediates signal transduction events and T-cell-B-cell interactions that regulate cell development, activation, growth, and motility [48]. The protein encoded by CD40 has the same function in the immune system and in inflammation. As a member of the TNF-receptor superfamily, the protein encoded by CD40 is associated with many diseases such as autoimmune disease, tissue inflammation, and so on [49]–[51]. The protein encoded by CXCR3 was reported to be expressed in activated T lymphocytes and natural killer (NK) cells [52]. A relationship between CXCR3 and atherosclerosis, type I diabetes, and acute cardiac allograft rejection has also been shown in previous studies [53]–[55]. The PSMB9 gene, which is located in the class II region of the major histocompatibility complex (MHC), was found to be related to a series of immune-related diseases [56]. IL7R, located on 5p13, has been shown to be associated significantly with infection, inflammation, and CAD. In 2011, Pickens et al. [57] suggested that IL7 and its receptor were overexpressed in rheumatoid arthritis synovial fluid and peripheral blood macrophages, as well as fibroblasts. Although significant correlations were found between the abovementioned genes and ED risk, after restricting the FWER value to 0.5, only the IL27 gene showed a significant association with ED. Thus, we suggest that IL27 may be one of the genes involved in the induction and regulation of ED.

On the basis of the relevance of the genes in infection, inflammation, diabetes mellitus, CAD, and so on, we propose that these immune-system-related genes might be associated with ED risk significantly, because they regulate cytokine homeostasis, which could induce a series of diseases. These genes, especially IL27, could provide new views on gene therapy.

### Limitations

This study has some limitations that need to be noted. (1) This cross-sectional study was based on a large population-based investigation. There were many inevitable restrictions, such as samples losses, incomplete information, as well as other problems. (2) Although we formulated rigorous standards for excluding and including participants in this analysis, we could not ensure that the confounding factors were excluded absolutely. (3) In this study, the International Index of Erectile Function (IIEF-5) was applied in order to identify ED, which could mix psychological and organic diseases, while increasing the bias in the results. So, further study was needed to confirm the research. (4) The samples in our study consisted solely of the serum, which could represent the systemic immune situation; however, the real circumstance in the organ was unclear. (5) There are many methods available for analyzing the association between genes and diseases, but each method carries a risk of providing a false-positive result. In this study, SKAT analysis was applied. Although this method could remedy the defects of traditional GWAS analysis and it could detect multi-SNP and epistatic effects, the results might be influenced by the selection of tag-SNPs, numbers of SNPs in each gene, and the powers of every SNP in the study. So, the genes identified by this method might not only be associated ED risk, but also other related diseases. (6) The absolute levels of identified gene products were not present to confirm these identified genes.

## Conclusions

ED is a prevalent disease that influences a large percentage of males and has a significant negative impact on the lives of patients and the economy. Considering the potential association of infection and low-grade inflammation with ED risk, we conducted a cross-sectional analysis to investigate the relationship between immune substances and ED. Our results suggest that IgG and seven genes (PTAFR, CD37, CD40, IL7R, PSMB9, CXCR3, and especially IL27) might be key factors in the pathogenesis of ED, which could pave the way for gene and immune therapies. The correlation is to be confirmed in further studies.

## Supporting Information

Table S1 **The primary data used in the study for C3.**
(XLS)

Table S2 **The primary data used in the study for C4.**
(XLS)

Table S3 **The primary data used in the study for IgA.**
(XLS)

Table S4 **The primary data used in the study for IgG.**
(XLS)

Table S5 **The primary data used in the study for IgM.** * Education. A01. 0–6∶1; 7–9∶2; ≥10∶3. * Smoke. B01∶1 smoke; 2 never smoke. B02∶1 current smoke; 2 smoking cessation. * Drink. C01∶1 drinking; 2 no drinking. * IIEF. D01: How do you rate your confidence that you could get and keep an erection?. D02: When you had erections with sexual stimulation, how often were your erections hard enough for penetration? D03: During sexual intercourse, how often were you able to maintain your erection after you had penetrated (entered) your partner? D04: During sexual intercourse, how difficult was it to maintain your erection to completion of intercourse? D05: When you attempted sexual intercourse, how often was it satisfactory for you?
(XLS)

## References

[1] National Institutes of Health (1993) Consensus development conference statement. Impotence. December 7–9, 1992. Int J Impot Res 5: 181–284. [PubMed: 8173631]. 8173631

[2] Hatzimouratidis K, Amar E, Eardley I, Giuliano F, Hatzichristou D, et al.. (2010) Guidelines on male sexual dysfunction: erectile dysfunction and premature ejaculation. Eur Urol 57: 804–814. [PubMed: 20189712]. 10.1016/j.eururo.2010.02.020 20189712

[3] Shah J (2002) Erectile dysfunction through the ages. BJU Int 90: 433–441. [PubMed: 12175404]. 10.1046/j.1464-410x.2002.02911.x 12175404

[4] Lewis RW, Fugl-Meyer KS, Corona G, Hayes RD, Laumann EO, etal. (2010) Definitions/epidemiology/risk factors for sexual dysfunction. J Sex Med 7: 1598–1607. [PubMed: 20388160]. 10.1111/j.1743-6109.2010.01778.x 20388160

[5] Braun M, Wassmer G, Klotz T, Reifenrath B, Mathers M, etal. (2000) Epidemiology of erectile dysfunction: results of the ‘Cologne Male Survey’. Int J Impot Res 12: 305–311. [PubMed: 11416833]. 10.1038/sj.ijir.3900622 11416833

[6] Pinnock CB, Stapleton AM, Marshall VR (1999) Erectile dysfunction in the community: a prevalence study. Med J Aust 171: 353–357. [PubMed: 10590723]. 10.5694/j.1326-5377.1999.tb123691.x 10590723

[7] Nicolosi A, Glasser DB, Kim SC, Marumo K, Laumann EO, etal. (2005) Sexual behaviour and dysfunction and help-seeking patterns in adults aged 40–80 years in the urban population of Asian countries. BJU Int 95: 609–614. [PubMed: 15705089]. 10.1111/j.1464-410X.2005.05348.x 15705089

[8] Nicolosi A, Moreira ED Jr, Shirai M, Bin Mohd Tambi MI, Glasser DB (2003) Epidemiology of erectile dysfunction in four countries: cross-national study of the prevalence and correlates of erectile dysfunction. Urology 61: 201–206. [PubMed: 12559296]. 10.1016/s0090-4295(02)02102-7 12559296

[9] Bacon CG, Mittleman MA, Kawachi I, Giovannucci E, Glasser DB, etal. (2003) Sexual function in men older than 50 years of age: results from the health professionals follow-up study. Ann Intern Med 139: 161–168. [PubMed: 12899583]. 10.7326/0003-4819-139-3-200308050-00005 12899583

[10] Ayta IA, McKinlay JB, Krane RJ (1999) The likely worldwide increase in erectile dysfunction between 1995 and 2025 and some possible policy consequences. BJU Int 84: 50–56. [PubMed: 10444124]. 10.1046/j.1464-410x.1999.00142.x 10444124

[11] Weinberg AE, Eisenberg M, Patel CJ, Chertow GM, Leppert JT (2013) Diabetes Severity, Metabolic Syndrome, and the Risk of Erectile Dysfunction. J Sex Med 10: 3102–3109. [PubMed: 24010555]. 10.1111/jsm.12318 PMC3891923 24010555

[12] Rendell MS, Rajfer J, Wicker PA, Smith MD (1999) Sildenafil for treatment of erectile dysfunction in men with diabetes: a randomized controlled trial. Sildenafil Diabetes Study Group. JAMA 281: 421–426. [PubMed: 9952201]. 10.1001/jama.281.5.421 9952201

[13] Dunsmuir WD, Holmes SAV (1996) The aetiology and management of erectile, ejaculatory, and fertility problems in men with diabetes mellitus. Diabetic Med 13: 700–708. [PubMed: 8862943]. 10.1002/(SICI)1096-9136(199608)13:8<700::AID-DIA174>3.0.CO;2-8 8862943

[14] Kumar J, Bhatia T, Kapoor A, Ranjan P, Srivastava A, etal. (2013) Erectile dysfunction precedes and is associated with severity of coronary artery disease among Asian Indians. J Sex Med 10: 1372–1379. [PubMed: 23347017]. 10.1111/jsm.12041 23347017

[15] Nehra A, Jackson G, Miner M, Billups KL, Burnett AL, etal. (2013) Diagnosis and treatment of erectile dysfunction for reduction of cardiovascular risk. J Urol 189: 2031–2038. [PubMed: 23313195]. 10.1016/j.juro.2012.12.107 23313195

[16] Blans MC, Visseren FL, Banga JD, Hoekstra JB, van der Graaf Y, etal. (2006) Infection induced inflammation is associated with erectile dysfunction in men with diabetes. Eur J Clin Invest 36: 497–502. [PubMed: 16796607]. 10.1111/j.1365-2362.2006.01653.x 16796607

[17] Araña Rosaínz Mde J, Ojeda MO, Acosta JR, Elías-Calles LC, González NO, etal. (2011) Imbalanced low-grade inflammation and endothelial activation in patients with type 2 diabetes mellitus and erectile dysfunction. J Sex Med 8: 2017–2030. [PubMed: 21554550]. 10.1111/j.1743-6109.2011.02277.x 21554550

[18] Lu Z, Gao Y, Tan A, Yang X, Zhang H, etal. (2012) Increased high-sensitivity c-reactive protein predicts a high risk of lower urinary tract symptoms in chinese male: Results from the fangchenggang area male health and examination survey. Prostate 72: 193–200. [PubMed: 21594882]. 10.1002/pros.21421 21594882

[19] Rosen RC, Cappelleri JC, Smith MD, Lipsky J, Peña BM (1999) Development and evaluation of an abridged, 5-item version of the International Index of Erectile Function (IIEF-5) as a diagnostic tool for erectile dysfunction. Int J Impot Res 11: 319–326. [PubMed: 10637462]. 10.1038/sj.ijir.3900472 10637462

[20] Kupelian V, Araujo AB, Chiu GR, Rosen RC, McKinlay JB (2010) Relative contributions of modifiable risk factors to erectile dysfunction: results from the Boston Area Community Health (BACH) Survey. Prev Med 50: 19–25. [PubMed: 19944117]. 10.1016/j.ypmed.2009.11.006 PMC2813912 19944117

[21] Liao M, Huang X, Gao Y, Tan A, Lu Z, etal. (2012) Testosterone is associated with erectile dysfunction: a cross-sectional study in Chinese men. PLoS One 7: e39234. [PubMed: 22737230]. 10.1371/journal.pone.0039234 PMC3380865 22737230

[22] Shiels MS, Rohrmann S, Menke A, Selvin E, Crespo CJ, etal. (2009) Association of cigarette smoking, alcohol consumption, and physical activity with sex steroid hormone levels in US men. Cancer Causes Control 20: 877–886. [PubMed: 19277882]. 10.1007/s10552-009-9318-y PMC3004151 19277882

[23] Chen C, Lu FC, Department of Disease Control Ministry of Health, PR China. (2004) The guidelines for prevention and control of overweight and obesity in Chinese adults. Biomed Environ Sci 17 Suppl: 1–36. [PubMed: 15807475]. 15807475

[24] Grundy SM, Cleeman JI, Daniels SR, Donato KA, Eckel RH, etal. (2005) Diagnosis and management of the metabolic syndrome: an American Heart Association/National Heart, Lung, and Blood Institute Scientific Statement. Circulation 112: 2735–2752. [PubMed: 16157765]. 10.1161/CIRCULATIONAHA.105.169404 16157765

[25] Tan A, Sun J, Xia N, Qin X, Hu Y, etal. (2012) A genome-wide association and gene-environment interaction study for serum triglycerides levels in a healthy Chinese male population. Hum Mol Genet 21: 1658–1664. [PubMed: 22171074]. 10.1093/hmg/ddr587 22171074

[26] Yang C, Jie W, Yanlong Y, Xuefeng G, Aihua T, etal. (2012) Genome-wide association study identifies TNFSF13 as a susceptibility gene for IgA in a South Chinese population in smokers. Immunogenetics 64: 747–753. [PubMed: 22864923]. 10.1007/s00251-012-0636-y 22864923

[27] Ortutay C, Vihinen M (2006) Immunome: a reference set of genes and proteins for systems biology of the human immune system. Cell Immunol 244: 87–89. [PubMed: 17434156]. 10.1016/j.cellimm.2007.01.012 17434156

[28] Xu Z, Taylor JA (2009) SNPinfo: integrating GWAS and candidate gene information into functional SNP selection for genetic association studies. Nucleic Acids Res 37(Web Server issue): W600–5. [PubMed: 19417063]. 10.1093/nar/gkp290 PMC2703930 19417063

[29] Purcell S, Neale B, Todd-Brown K, Thomas L, Ferreira MA, etal. (2007) PLINK: a tool set for whole-genome association and population-based linkage analyses. Am J Hum Genet 81: 559–575. [PubMed: 17701901]. 10.1086/519795 PMC1950838 17701901

[30] Wu MC, Kraft P, Epstein MP, Taylor DM, Chanock SJ, etal. (2010) Powerful SNP-set analysis for case-control genome-wide association studies. Am J Hum Genet 86: 929–942. [PubMed: 20560208]. 10.1016/j.ajhg.2010.05.002 PMC3032061 20560208

[31] Feeley RJ, Traish AM (2009) Obesity and erectile dysfunction: is androgen deficiency the common link? ScientificWorldJournal 9: 676–684. [PubMed: 19649506]. 10.1100/tsw.2009.79 PMC5823217 19649506

[32] Glina S, Sharlip ID, Hellstrom WJ (2013) Modifying risk factors to prevent and treat erectile dysfunction. J Sex Med 10: 115–119. [PubMed: 22971247]. 10.1111/j.1743-6109.2012.02816.x 22971247

[33] Thorve VS, Kshirsagar AD, Vyawahare NS, Joshi VS, Ingale KG, etal. (2011) Diabetes-induced erectile dysfunction: epidemiology, pathophysiology and management. J Diabetes Complications 25: 129–136. [PubMed: 20462773]. 10.1016/j.jdiacomp.2010.03.003 20462773

[34] Shi MD, Chao JK, Ma MC, Chiang SK, Chao IC (2014) The connection between type 2 diabetes and erectile dysfunction in Taiwanese aboriginal males. Int J Impot Res. [PubMed: 25078051]. 10.1038/ijir.2014.26 25078051

[35] Cander S, Coban S, Altuner S, Oz Gul O, Yetgin ZA, et al.. (2014) Prevalence and correlates of erectile dysfunction in type 2 diabetes mellitus: a cross-sectional single-center study among Turkish patients. Metab Syndr Relat Disord. 12: 324–9. [PubMed: 24666397]. 10.1089/met.2013.0150 24666397

[36] Ettala OO, Syvänen KT, Korhonen PE, Kaipia AJ, Vahlberg TJ, et al.. (2014) High-Intensity Physical Activity, Stable Relationship, and High Education Level Associate with Decreasing Risk of Erectile Dysfunction in 1,000 Apparently Healthy Cardiovascular Risk Subjects. J Sex Med. [PubMed: 24909644]. 10.1111/jsm.12618 24909644

[37] Zwaka TP, Hombach V, Torzewski J (2001) C-reactive protein-mediated low density lipoprotein uptake by macrophages. Circualtion 103: 1194–1197. [PubMed: 11238260]. 10.1161/01.cir.103.9.1194 11238260

[38] Pasceri V, Willerson JT, Yeh ETH (2000) Direct proinflammatory effect of C-reactive protein on human endothelial cells. Circulation 102: 2165–2168. [PubMed: 11056086]. 10.1161/01.cir.102.18.2165 11056086

[39] Yin GN, Kim WJ, Jin HR, Kwon MH, Song KM, etal. (2013) Nerve injury-induced protein 1 (Ninjurin-1) is a novel therapeutic target for cavernous nerve injury-induced erectile dysfunction in mice. J Sex Med 10: 1488–1501. [PubMed: 23551591]. 10.1111/jsm.12129 23551591

[40] Honda Z, Ishii S, Shimizu T (2002) Platelet-activating factor receptor. J Biochem 131: 773–779. [PubMed: 12038971]. 10.1093/oxfordjournals.jbchem.a003164 12038971

[41] Koga MM, Bizzarro B, Sá-Nunes A, Rios FJ, Jancar S (2013) Activation of PAF-receptor induces regulatory dendritic cells through PGE2 and IL-10. Prostaglandins Leukot Essent Fatty Acids 89: 319–326. [PubMed: 24120121]. 10.1016/j.plefa.2013.09.003 24120121

[42] Stephen J, Emerson B, Fox KA, Dransfield I (2013) The Uncoupling of Monocyte-Platelet Interactions from the Induction of Proinflammatory Signaling in Monocytes. J Immunol 191: 5677–5683. [PubMed: 24133165]. 10.4049/jimmunol.1301250 24133165

[43] Predescu S, Knezevic I, Bardita C, Neamu RF, Brovcovych V, etal. (2013) Platelet Activating Factor-Induced Ceramide Micro-Domains Drive Endothelial NOS Activation and Contribute to Barrier Dysfunction. PLoS One 8: e75846. [PubMed: 24086643]. 10.1371/journal.pone.0075846 PMC3785431 24086643

[44] Lugg JA, González-Cadavid NF, Rajfer J (1995) The role of nitric oxide in erectile function. J Androl 16: 2–4. [PubMed: 7539414]. 7539414

[45] Larousserie F, Charlot P, Bardel E, Froger J, Kastelein RA, etal. (2006) Differential effects of IL-27 on human B cell subsets. J Immunol 176: 5890–5897. [PubMed: 16670296]. 10.4049/jimmunol.176.10.5890 16670296

[46] Villarino AV, Huang E, Hunter CA (2004) Understanding the pro- and anti-inflammatory properties of IL-27. J Immunol 173: 715–720. [PubMed: 16670296]. 10.4049/jimmunol.173.2.715 15240655

[47] Bosmann M, Ward PA (2013) Modulation of inflammation by interleukin-27. J Leukoc Biol 94: 1159–1165. [PubMed: 23904441]. 10.1189/jlb.0213107 PMC3828601 23904441

[48] van Spriel AB, Puls KL, Sofi M, Pouniotis D, Hochrein H, etal. (2004) A regulatory role for CD37 in T cell proliferation. J Immunol 172: 2953–2961. [PubMed: 14978098]. 10.4049/jimmunol.172.5.2953 14978098

[49] Huber AK, Finkelman FD, Li CW, Concepcion E, Smith E, etal. (2012) Genetically driven target tissue overexpression of CD40: a novel mechanism in autoimmune disease. J Immunol 189: 3043–3053. [PubMed: 22888137]. 10.4049/jimmunol.1200311 PMC3436983 22888137

[50] Guo CA, Kogan S, Amano SU, Wang M, Dagdeviren S, etal. (2013) CD40 deficiency in mice exacerbates obesity-induced adipose tissue inflammation, hepatic steatosis, and insulin resistance. Am J Physiol Endocrinol Metab 304: E951–63. [PubMed: 23482447]. 10.1152/ajpendo.00514.2012 PMC3651645 23482447

[51] Zhang X, Burch E, Cai L, So E, Hubbard F, etal. (2013) CD40 mediates downregulation of CD32B on specific memory B cell populations in rheumatoid arthritis. J Immunol 190: 6015–6022. [PubMed: 23686494]. 10.4049/jimmunol.1203366 23686494

[52] Qin S, Rottman JB, Myers P, Kassam N, Weinblatt M, etal. (1998) The chemokine receptors CXCR3 and CCR5 mark subsets of T cells associated with certain inflammatory reactions. J Clin Invest 101: 746–754. [PubMed: 9466968]. 10.1172/JCI1422 PMC508621 9466968

[53] Mach F, Sauty A, Iarossi AS, Sukhova GK, Neote K, etal. (1999) Differential expression of three T lymphocyte-activating CXC chemokines by human atheroma-associated cells. J Clin Invest 104: 1041–1050. [PubMed: 10525042]. 10.1172/JCI6993 PMC408576 10525042

[54] Frigerio S, Junt T, Lu B, Gerard C, Zumsteg U, etal. (2002) Beta cells are responsible for CXCR3-mediated T-cell infiltration in insulitis. Nat Med 8: 1414–1420. [PubMed: 12415259]. 10.1038/nm1202-792 12415259

[55] Hancock WW, Lu B, Gao W, Csizmadia V, Faia K, etal. (2000) Requirement of the chemokine receptor CXCR3 for acute allograft rejection. J Exp Med 192: 1515–1520. [PubMed: 11085753]. 10.1084/jem.192.10.1515 PMC2193193 11085753

[56] Yu L, Li Q, Lin J, Yu J, Li Q, etal. (2013) Association between polymorphisms of PSMB8, PSMB9 and TAP2 genes with rheumatoid arthritis in ethnic Han Chinese from Yunnan. Zhonghua Yi Xue Yi Chuan Xue Za Zhi 30: 222–226. [PubMed: 23568741]. 10.3760/cma.j.issn.1003-9406.2013.04.023 23568741

[57] Pickens SR, Chamberlain ND, Volin MV, Pope RM, Talarico NE, etal. (2011) Characterization of interleukin-7 and interleukin-7 receptor in the pathogenesis of rheumatoid arthritis. Arthritis Rheum 63: 2884–2893. [PubMed: 21647866]. 10.1002/art.30493 PMC3614067 21647866

